# EEG-ECeG Coherence Mapping of Human Cerebro-cerebellar Projections: An Exploratory Study

**DOI:** 10.1007/s12311-026-02065-4

**Published:** 2026-08-20

**Authors:** Neil P. M. Todd, Sendhil Govender, Daniel Hochstrasser, Peter E. Keller, James G. Colebatch

**Affiliations:** 1 UNSW Clinical School, Randwick Campus, Sydney, NSW 2052 Australia; 2 Department of Psychology, University of Exeter, Exeter, EX4 4QC UK; 3 Neuroscience Research Australia, UNSW, Sydney, NSW 2052 Australia; 4 MARCS Institute for Brain, Behaviour and Development, Western Sydney University Penrith, Sydney, NSW 2751 Australia; 5 Center for Music in the Brain, Department of Clinical Medicine, Aarhus University, Aarhus, 8000 Denmark

**Keywords:** Cerebellum, Cerebro-cerebellar tracts, Movement related coherence

## Abstract

The cerebellar and cerebral cortices are powerfully connected via reciprocal, crossed projections which mediate their coordination in motor, cognitive and affective processes. In the present paper we demonstrate non-invasive imaging of crossed cerebro-cerebellar connectivity by means of wavelet coherence. In a sample of six healthy adult subjects, we recorded EEG and the electro-cerebellogram (ECeG) with a 10% cerebellar extension montage during voluntary left and right index finger movements. EMG was also recorded from finger extensors and flexors and was used to generate triggers for movement related averaging (-2000 to + 2000 ms). Consistent with prior observations of movement related changes in delta/theta power, we observed significant movement related change in delta/theta-band EEG-ECeG coherence between cerebral and cerebellar leads. Of particular note, when lateralised seeds were selected (F1 vs. F2 and SO11 vs. SO12) the low-frequency coherence was distributed contralaterally and frontally for cerebellar seeds, likely reflecting the underlying crossed cerebro-cerebellar projections, as well as the cerebral contralateral somatosensory representation. For the F1/F2 seeds the movement related change in delta/theta-band EEG-ECeG coherence was more bilateral but with right-hemisphere dominance. The cerebral frontally seeded coherence further demonstrated strong cortico-cortical delta/theta coherence with bilateral posterior parietal and temporal cortex, as well as beta/gamma coherence with primary sensory-motor cortex for the hands. These preliminary findings further support the value of recording cerebellar ECeG and demonstrate its potential to contribute to the understanding of cerebro-cerebellar function and dysfunction.

**Supplementary Information:**

The online version contains supplementary material available at https://doi.org/10.1007/s12311-026-02065-4.

## Introduction

It is generally accepted that there are two dominant sensori-motor homunculi in the human cerebellum, one inverted in the anterior lobe and a second located in the posterior lobe [1–4]. Both cerebellar homunculi are predominantly ipsilateral to the corresponding body parts and contralateral to the associated cerebral homunculi in the sensory-motor cortex, connected bidirectionally via the crossed cortico-ponto-cerebellar and cerebello-thalamo-cortical tracts. This system is one of the largest in the central nervous system involving in addition the topographical and temporal coordination of the cortico-ponto-cerebellar and cerebro-olivo-cerebellar systems, allowing precise convergence of mossy and climbing fibre inputs within cerebellar modules from a common cerebral origin [5].

Cerebro-cerebellar connectivity has been studied in animal models using tract tracing [6] and in vivo in humans using tractographic methods [7, 8], whole-brain structural covariation modeling [9], and resting-state [10] and task-based functional connectivity analyses [11]. In addition to motor connectivity these have revealed wide-spread activity in non-motor areas, including prefrontal and posterior parietal cortex [12, 13]. While these approaches have provided detailed maps of interaction pathways linking the cerebellum and cerebral cortex, physiological interaction patterns along these pathways can be examined dynamically and in a frequency-specific manner using coherence analysis. Such studies using intra-cortical recordings have demonstrated coherence over a range of frequencies from delta (*δ*), through theta (*θ*), alpha (*α*), beta (*β*) and gamma (*γ*) bands which can be modulated by behaviour such as during grasping [14–19].

EEG and MEG coherence have long been employed for the study of long-range neural connectivity between areas of the cerebral cortex, e.g. [20–27]. A few studies have recorded MEG combined with source analysis for cerebro-cerebellar coherence in humans during behaviour [28, 29]. However, it had been generally thought that it was not possible to record directly from the cerebellum by EEG/MEG due to noise sources from the neck [30].

Recent developments in non-invasive electrophysiology have shown that, contrary to prior belief, the posterior lobe at least is within the range of EEG and MEG sensors [31, 32]. In our prior work we have shown that it is possible to record both cerebellar evoked potentials (CEPs) and the spontaneous activity of the cerebellum, the electrocerebellogram (ECeG). The latter characteristically contains much higher frequency content than EEG [32–34]. It is of interest, therefore, to determine whether it is possible to record cerebro-cerebellar coherence that reliably reflects known physiological interaction pathways.

The present study is the fourth analysis arising from a common EEG-ECeG dataset acquired during self-paced voluntary movement. Previous studies based on these recordings examined (i) the source localisation and physiological origins of the underlying movement-related potentials [35], (ii) movement-related changes in EEG and ECeG spectral power [36], and (iii) the feasibility of decoding cerebellar movement-related potentials for brain-computer interface applications [37]. The present work extends this programme through an exploratory analysis of EEG-ECeG coherence, with the aim of identifying functional cerebro-cerebellar interactions associated with voluntary movement. We focus here on established pathways associated with somatotopic sensorimotor representations of the hands. Our study specifically examines modulations of coherence as a marker of stages in the preparation and execution of self-initiated finger movements in the form of movement related changes in power, i.e. movement related desynchronisation (MRD) or synchronisation (MRS), and associated movement related coherence (MRC) [35, 36]. We do so by conducting four coherence mapping studies starting with seeds located close to the cerebral sensorimotor representations of the hands, i.e. at central electrode sites C3 and C4, then tracing focal points at frontal, cerebellar and parietal sites during rest and then during left versus right hand movement. Although our primary interest is in cerebro-cerebellar pathways, the exploratory nature of the coherence-mapping approach necessitates placing the specific cerebro-cerebellar findings within the broader network architecture underlying self-initiated movement.

## Methods

### Subjects

Six healthy adult subjects (5 males, 1 female) were recruited from staff and students (age range 25–65) at the Prince of Wales Hospital, the University of Western Sydney and the University of Melbourne, as for [36, 37]. All were right handed (self-report). All subjects gave written informed consent, and the study was approved by the local ethics committee (South Eastern Sydney Local Health District Human Research Ethics Committee). All procedures were carried out in accordance with the Declaration of Helsinki.

### EEG/ECeG Recording

**Fig. 1:**
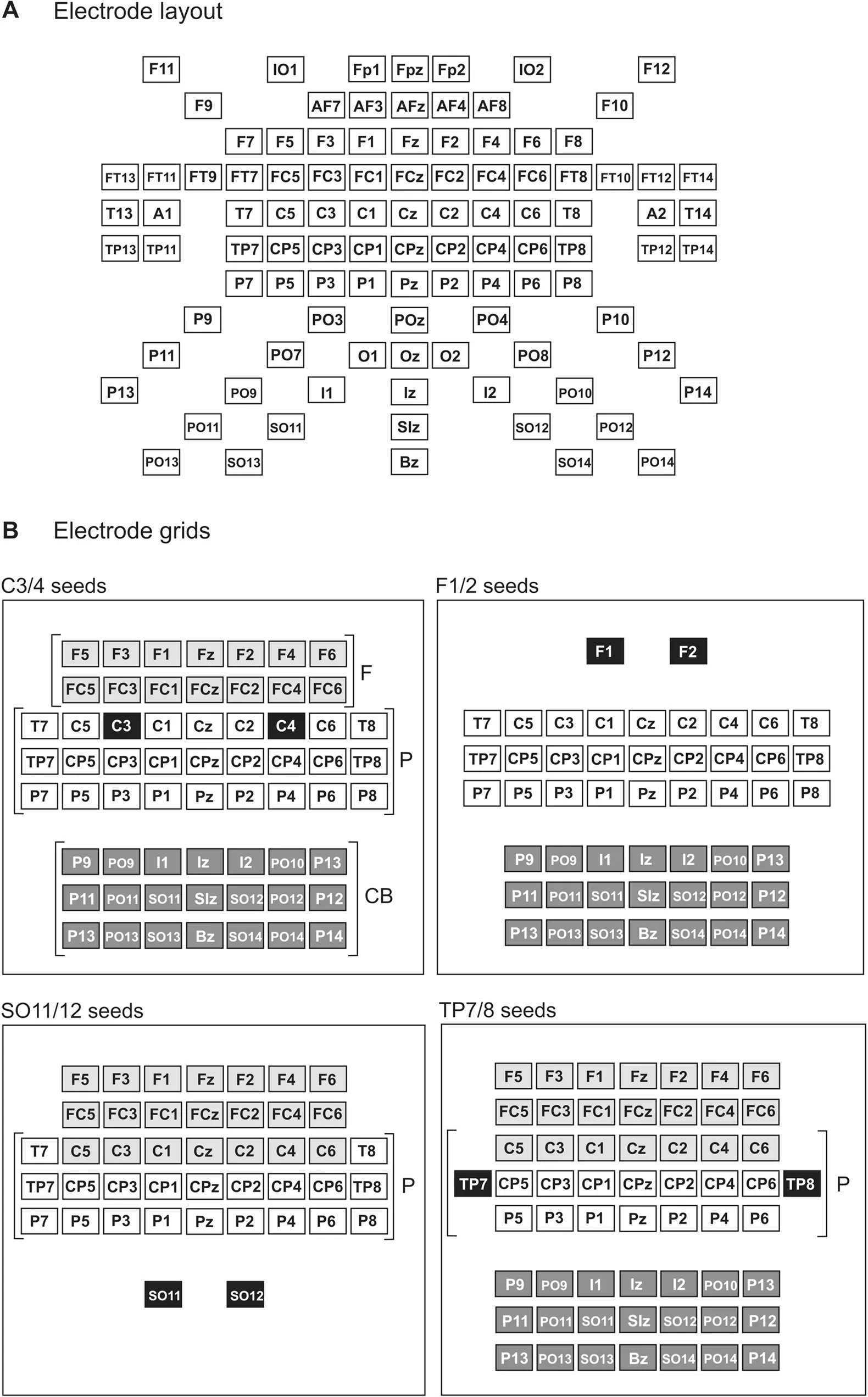
(**A**) Spatial layout of the 102 cephalic electrodes used during EEG recordings. An additional electrode pair was placed bilaterally over the SCM muscles (not shown), giving a total of 104 recording channels. (**B**) Electrode grids employed for statistical analysis with each of the four seed-pairs employed. (Top left) C3/4, (top right) F1/2, (bottom left) SO11/12 and (bottom right) TP7/8. F – Frontal, P –Parietal, CB – cerebellar. For F1/F2 and SO11/12 seeds, local electrode comparisons were not included in the analyses

**Fig. 2:**
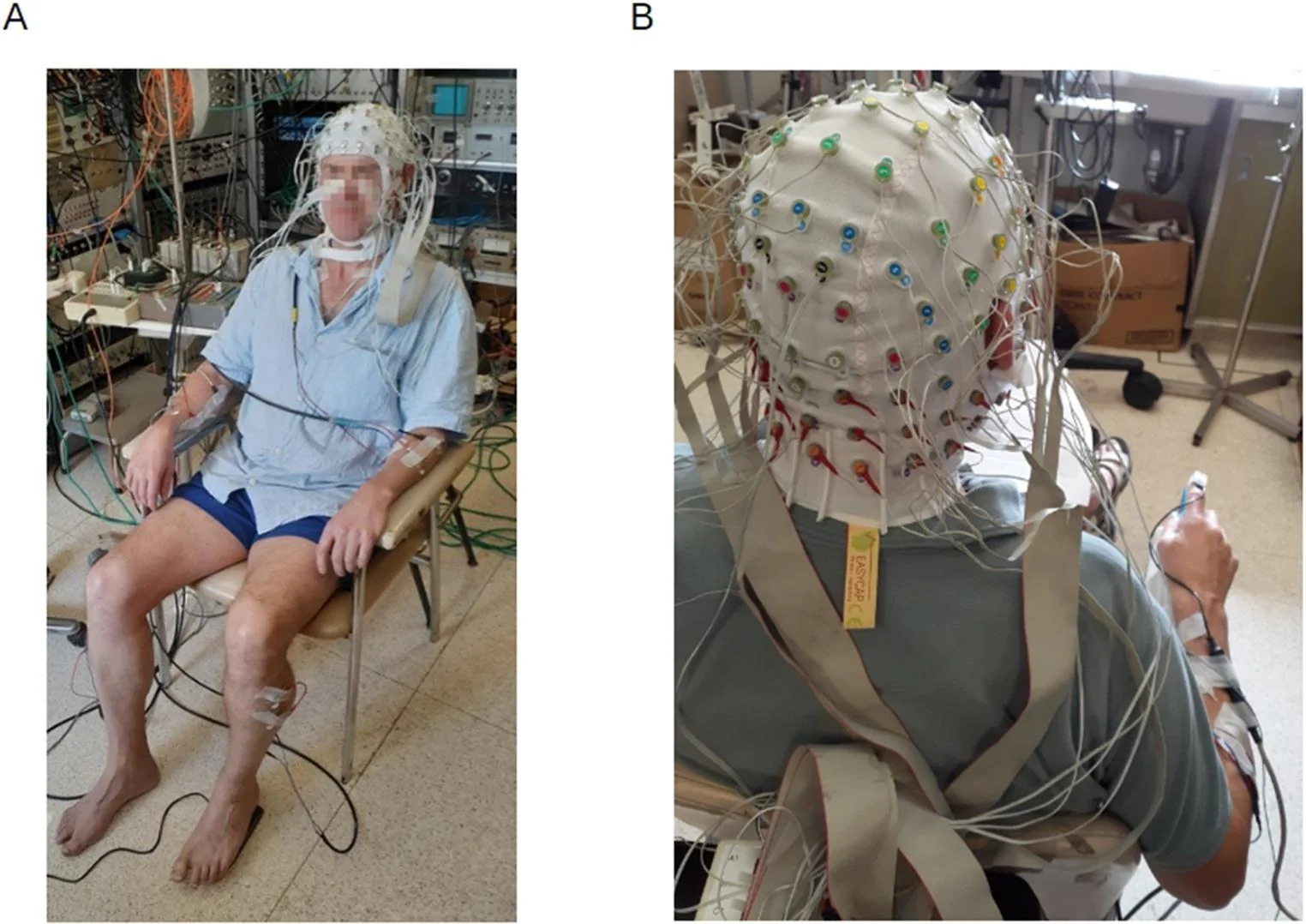
Photographs illustrating the experimental set-up. (**A**) A volunteer from the front with 10% cerebellar extension EEG cap and EMG recording electrodes for left and right upper and lower limb extensors and flexors. (**B**) A volunteer from behind showing detail of the cerebellar extension electrode locations and accelerometer placement on the right index finger

EEG was recorded using a 104 (96 cap + 8 external, EasyCap GmbH) channel custom 10% cerebellar extension montage designed by completing the population of the Iz circle, and then two additional 10% rows below (Figs. 1 and 2), as described in [38]. ActiveTwo amplifiers (BioSemi, Holland) were used. 102 of the 104 channels were from cephalic electrode sites, including a pair of electrodes placed infra-ocularly (IO), with the last pair on the sternocleidomastoid (SCM) muscle. The sampling rate was 4096 Hz.

### EMG Recording and Triggering

For recording finger extension EMG, bipolar electrode pairs (Cleartrace 1700-030, Conmed Corp., USA) were located to the extensor indices (EI) and flexor digitorum muscles from the left and right hands (Fig. 2). For foot dorsiflexion EMG bipolar electrode pairs were located to tibialis anterior (TA) and soleus (SOL) from left and right legs (Fig. 2). EMG signals were amplified (x1000, Medelec AA6, Mark III), band-pass filtered (2 Hz − 1.6 kHz), sampled at 10 kHz using a Power1401 and Signal software (6.1, CED Electronics, UK). Rectified EMG signals from the target muscles (EI, for finger extension, TA for foot dorsiflexion) were thresholded and debounced for the production of a trigger to signal movement onset. The Power1401 digital output sent a 1 ms pulse to the BioSemi trigger box to indicate movement onset.

### Movement Task, Accelerometry and Foot Displacement Measurement

As described in Todd et al. [35], for each subject, recordings were made under five conditions. (1) Initially, subjects were required to sit at rest for 5 min with eyes in a fixed neutral position ahead. In four subsequent recording sessions, subjects, after suitable training to minimise EMG/EOG artefact, were required: (2) to extend the right index finger; (3) to extend the left index finger; (4) to dorsiflex the right foot; and (5) to dorsiflex the left foot. The present analysis focused on the upper-limb conditions only because the cerebral somatotopic representations of the hands are more readily accessible from surface recordings. Additional training of about 10 trials with feedback was given to ensure a sufficiently brisk movement at the required rate, about once every 5 s, which would reliably produce a trigger. This training session also allowed the adjustment of the threshold to an appropriate level. For each of the movement tasks a unilateral accelerometer (model 3026-200-S, ICSensors, USA) was used to record finger and foot acceleration, amplified (x1000, custom bridge amplifier) and sampled at 10 kHz using the CED Power1401. The accelerometer was secured on the distal phalanx during right and left index finger extension and on the dorsum of the foot during dorsiflexion. For foot dorsiflexion, angular displacement was also recorded by means of a custom adapted sewing machine foot controller (model ES01FC, Shanrya). Subjects were asked to keep their gaze forward throughout to minimise eye and head movement. 100 trials were recorded for each condition. Subjects were given a break at 50 trials. A small number of trials (two to three) were lost due to error in some cases.

### EEG/ECeG Screening and Averaging

Using Brain Electrical Source Analysis (BESA (version 7.1, MEGIS Software GmbH, Germany), EEG/ECeG recordings were initially screened for blinks, eye movement and ECG artifacts. For most small eye blink/movement artefacts, the BESA artefact removal algorithm was employed along with the ECG artefact removal algorithm after modelling the ECG by a single dipole in the neck, with the left and right SCM channels used to detect ECG. The EEG/ECeG was then epoched to − 2000 ms to + 2000 ms around the EMG trigger and the baseline correction set to − 2000 ms to − 1500 ms. A high-pass filter of 0.2 Hz was applied with forward phase at 6 dB/octave roll-off. After the individual mean MRPs were produced for each of the subjects, a small number of channels that were noisy due to residual artefact were interpolated [35, 36]. To eliminate the effects of mains contamination, a narrow band-stop filter at 50 Hz and all harmonics to 650 Hz was applied prior to analysis.

### Coherence Analysis

After recording EMG/EEG/ECeG and epoching (− 2 s to + 2 s), we conducted a coherence analysis on all EEG/ECeG channels, with specific seed/hand combinations (e.g. C3/right hand, or C4/left hand) as described in the “mapping” section below, using the wavelet cross-spectrum (WCS) as implemented in the MATLAB toolbox (R2019b, Mathworks, Natick, CA).

The WCS is a measure of shared power between two time series *a*_*n*_ and *b*_*n*_, where *n* = {1…*N*} is sample number with *N* the total number of samples. If *A*_*n, v*_ and *B*_*n, v*_ are the respective continuous wavelet transforms of the two series (two-dimensional complex arrays using the Morlet analysing wavelet), where *v* = {1…*V*} is voice number in a scale/frequency-space with *V* the total number of voices, then the WCS is defined as

$$\:{AB}_{n,v}={A}_{n,v}\cdot\:{{B}^{*}}_{n,v}$$

where B*_n, v_ is the complex conjugate of *B*_*n, v*_. In the MATLAB implementation a smoothing operator is also applied to the product. The wavelet coherence *C*_*n, v*_(*a*,* b*) between time series *a*_*n*_ and *b*_*n*_, as employed in Todd et al. [34], is related to the cross-spectrum by

$$\:C{{\left(a,b\right)}^{2}}_{n,v}\mathrm{=}\frac{{\left|{AB}_{n,v}\right|}^{2}}{{\left|{A}_{n,v}\right|}^{2}\cdot\:{\left|{B}_{n,v}\right|}^{2}},$$

and is real valued between 0 and 1. For our current purpose, however, the WCS has the great advantage within the MATLAB implementation that it is possible to extract the phase by separation of the real and imaginary components. In the present analyses the *AB*_*n, v*_ were computed over the whole 4 s epoch (thus *N* = 4096*4 = 16, 384) for each trial of each seed/target pair and averaged as complex objects over all trials (up to 100). Thus if *m* = {1...*M*} is the trial number and *M* the total number of trials, for each subject and each seed/target pair $\:\overline{{AB}_{n,v}}\mathrm{=}{{\sum\nolimits^{M}_{m=1}}}\frac{{AB}_{m,n,v}}M$. The WCS was employed at a density of 24 voices per octave over 9 octaves (thus *V* = 24*9 + 1 = 217). Within the MATLAB implementation for the sampling rate of 4096 Hz and epoch length of 4 s, this spans a frequency range of about 0.5 Hz to 2000 Hz. The WCS were then split into five frequency bands: delta (*δ*: 1.5 –3 Hz), theta (*θ*: 3–6 Hz), alpha (*α*: 6–12 Hz), beta (*β*: 13–30 Hz) and gamma (*γ*: 30–80 Hz). The lower and higher frequency bands were not employed here. These were then further segmented into 16 equal time segments to allow for ANOVA testing of movement related changes. The segmentation strategy was also similar to that of Todd et al. [36] and chosen to equally represent pre- and post-movement epochs (− 1.5 to + 1.5 s), but exclude the epoch ends to remove filter end-effects.

To avoid artefactual self-coherence due to volume conduction and reference effects, after averaging within subjects, only the imaginary component of the WCS, i.e. Im($\:\overline{{AB}_{n,v}}$), was employed for across subject averaging and statistics [39, 40]. The averaged Im($\:\overline{{AB}_{n,v}}$) across subjects was also used to generate the images (coherograms or cross-scaleograms) for the coherence maps. For the statistical analysis, for any single frequency band, for each subject the within-band Im($\:\overline{{AB}_{n,v}}$) values were averaged across total within-band voices, i.e. to obtain Im($\:\overline{\overline{{AB}_{n}}}$), before segmentation. Thus for any particular band and any particular combination of seed and target electrode, the within-subject data would be coded as a SUBJECT by SEGMENT array.

### Statistical Analysis

Statistical analyses of the frequency banded and time segmented imaginary component of the WCS were conducted by means of repeated measures analysis of variance (ANOVA) based on 5, 7 or 9 (“X”, lateral) by 3 (“Y”, anteroposterior) grids centred on either FCz (“frontal”), CPz (“parietal”) or SIz (“cerebellar”). The motivation for the choice of the ANOVA grid centred at FCz was because this corresponded to where the maximal power associated with the underling BPs was located, as shown in Todd et al. [36], i.e. including motor, premotor and supplementary motor area (SMA) sources. Similarly the ANOVA grid at SIz would cover the cerebellar posterior motor projection areas. The grid at CPz would cover areas for both fronto-parietal and cerebello-parietal projection areas. For each of the three grid locations, within-subjects factors were HAND (Left vs. Right), “X” (1 to 5, 7 or 9), “Y” (1 to 3) and SEGMENT (1 to 16). Different X-ranges were employed as the spatial distribution of coherence is variable depending on the SEED-TARGET combination. The frontal grid tended to be focused towards the mid-line, reflecting the underlying Bereitschaftspotential sources. The parietal grid tended to be focused away from the mid-line. The cerebellar grid was more evenly distributed. If we were unsure we ran alternatives and report two alternatives for the SIz-FCz case.

With a choice of four seed locations, between two to three target grids and five frequency bands our statistical analysis would result in 55 separate ANOVA runs. Each repeated measures ANOVA employed four within subjects factors, resulting in 15 separate tests – four main effects and 11 interactions. Traditionally, a single or small number of ANOVA runs would not require correction for multiple comparisons and the 15 within ANOVA tests treated as independent. For a larger number of runs the risk of a chance false positive increases. A strict Bonferroni approach would, however, likely result in a complete loss of power to detect true positives since the total number of tests here is 55 * 15 = 825. An alternative less conservative approach is the false discovery rate (FDR) procedure [41]. As our study was exploratory we do not provide corrected p-values but do provide an estimate of likely FDR at three levels, *q* = 0.05, 0.1 and 0.2. Effects which fall outside these levels are treated as trend effects. In all cases, *p*-values were corrected for violations of sphericity using the Greenhouse-Geisser method.

### General Mapping Procedure

The total number of possible reciprocal seed-target combinations using the cerebellar extension montage with 96 channels is very high, being equal to 96!/(2!*94!) = 4560. Multiplying by movement conditions, rest, left and right hand, the number rises to 13,680. For this reason, we restricted the initial search for potential cerebellar motor/premotor maps by exploring the effect of cerebral central/frontal seed (hence ANOVA factor of SEED) locations. In particular, given the well-established approximate electrode location over the cerebral sensorimotor homunculus at C3 for the right hand (RH) and C4 for left hand (LH) this was the obvious starting point. We then generated maps for “at rest” and then L/RH movement in order to attempt to localise any reciprocal crossed hand area in the cerebellar leads, consistent with the known crossed cerebro-cerebellar projections. Subsequent choices were determined by distal focal points close to regions of interest, i.e. premotor cortex/SMA, posterior/inferior cerebellar motor and posterior parietal cortex.

## Results

In what follows we describe the details of mapping analyses for four choices of SEED pair electrodes (with four corresponding ANOVA tables). The procedure for choice of SEED was to start over the known cerebral sensorimotor areas for the left and right hands, i.e. at C3/C4 (“Coherence Maps with Sensorimotor Area Seeds C3/C4” section), create coherence maps and then locate apparent distal focal sites within these, close to regions of interest. The maps created by the C3/C4 SEEDs suggested distal focal points at F1/F2, close to premotor cortex/SMA, and these were then selected for the 2nd maps (“Coherence Maps with Frontal Seeds F1/2” section). The maps created from the F1/F2 SEEDs then in turn suggested other distal focal points, including at SO11/SO12, close to the expected location of the posterior cerebellar sensorimotor homunculus (“Coherence Maps with Cerebellar Seeds SO11/12” section). The maps created from the SO11/SO12 SEEDs, as well as apparent reciprocal focal sites near F1/F2 also appeared to show focal sites at lateral posterior parietal positions, hence the choice of TP7/TP8 SEEDs (“Coherence Maps with Posterior Parietal Seeds TP7/TP8” section). For the “at rest” conditions the ANOVA SEED factor was uniquely defined. For the movement conditions the factors SEED and HAND collapsed to a single SEED/HAND factor, e.g. C3/RH versus C4/LH. In the final “False Discovery Rate (FDR) analysis” section we attempt to provide a summary analysis of general observations from the four mapping analyses.

### Coherence Maps with Sensorimotor Area Seeds C3/C4

#### Rest Effects

**Fig. 3:**
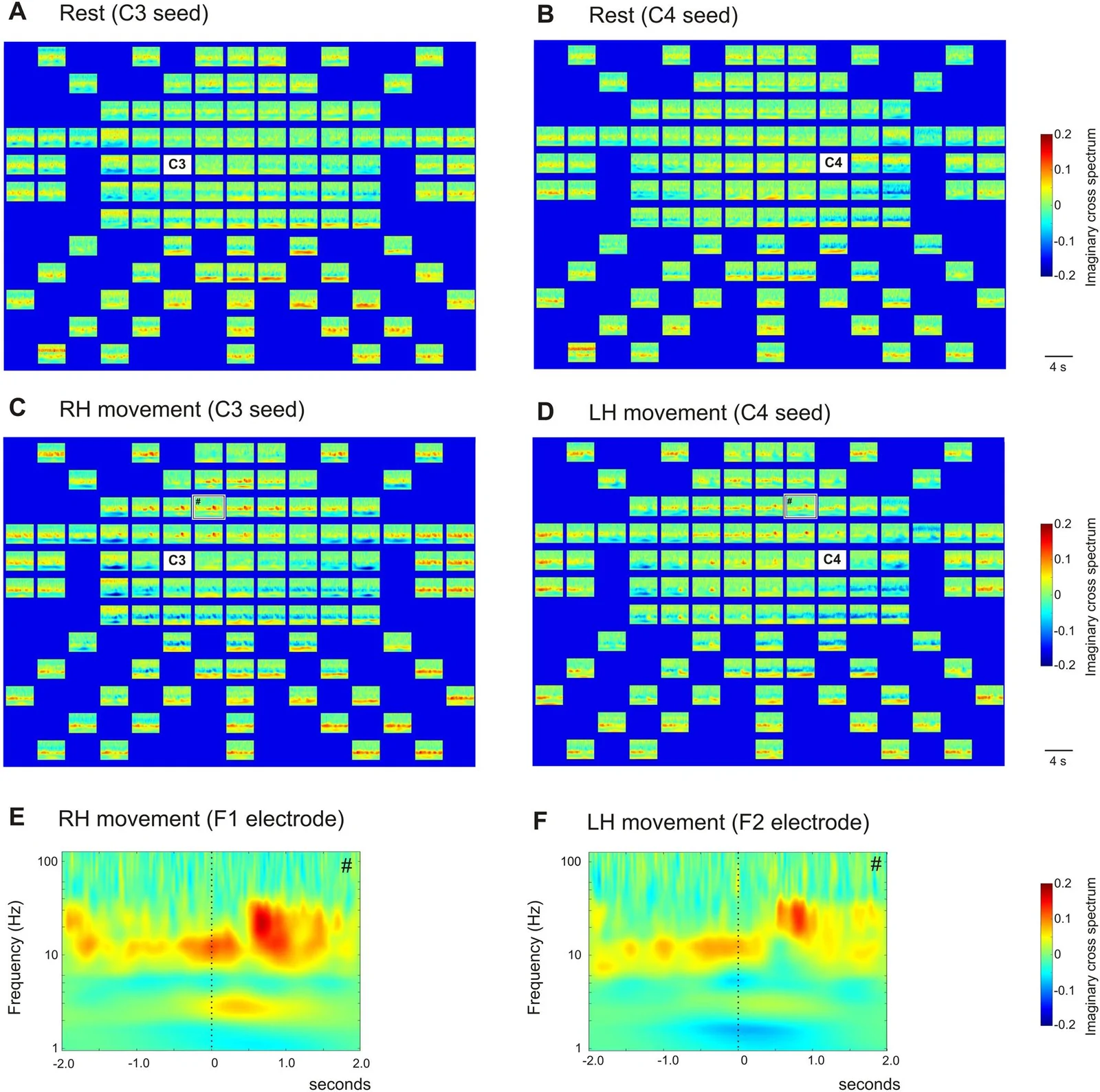
Coherence maps during rest and movement conditions using central seeds (C3/C4). Each panel displays the time–frequency imaginary cross-spectrum between the seed electrode and a single scalp electrode. The top row shows mappings using C3 (**A**) and C4 (**B**) as seeds during rest. The middle row presents coherence maps during right-hand (RH) and left-hand (LH) movements using C3 (**C**) and C4 (**D**) as seeds, respectively. The bottom row shows single-electrode maps at the frontal sites; F1 (E; RH movement) and F2 (F; LH movement)

Figure 3 A/B illustrates the maps produced at C3/C4 at rest. The resting coherence maps are generally homogenous, although they do show some evidence of a standing distribution of *δ/θ*-coherence in frontal, parietal and cerebellar sites, manifest in terms of X and Y ANOVA effects. For the frontal sites, excluding the Cz row, this is symmetrical about the positive phase midline (*δ−*band, *F*(6,30) = 3.8, *p* < .05). For the parietal sites, including the Cz row, it is also symmetrical about the positive phase midline and posteriorly (respectively for *δ−*band X and Y effects, *F*(8,40) = 5.3, *p* < .05 and *F*(2,10) = 8.7, *p* < .05 and for *δ/θ*-band X*Y effects *F*(16,80) = 3.9, *p* < .05 and *F*(16,80) = 6.6, *p* < .05). For the cerebellar sites the standing distribution of *δ−*coherence is generally skewed to the right hemisphere and anteriorly, manifest as an X*Y interaction (*F*(12,60) = 4.6, *p* < .05). There were no SEED or SEGMENT effects at rest.

#### Delta/theta-band Movement Effects

**Fig. 4:**
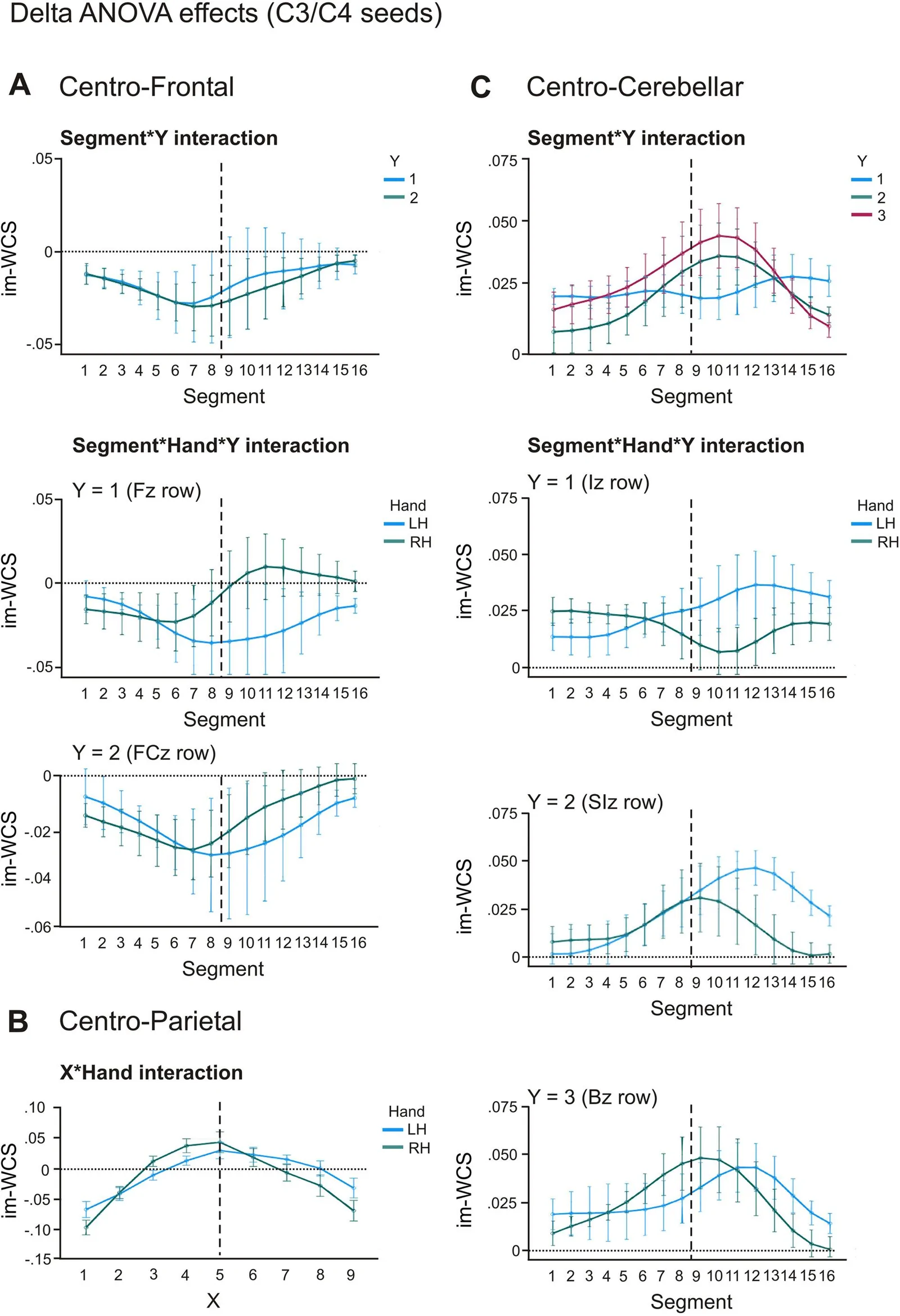
Changes in delta coherence with C3/4 seeds. ANOVA effects are shown for changes in centro-frontal (**A**), centro-parietal (**B**) and centro-cerebellar (**C**) coherence. In (A) Y = 1–2 refers to the Fz (midline frontal) and FCz (midline fronto-central) rows; in (B) X = 1–9 refers to the TP7, CP5, CP3, CP1, Cpz, CP2, CP4, CP6 and TP8 columns; in (C) Y = 1–3 refers to the Iz, SIz and Bz rows. Points represent estimated marginal means from the repeated-measures ANOVA. Error bars indicate ± 1 SEM; *n* = 6 participants

Figure 3 C/D respectively display the coherence maps for right and left hand movement respectively seeded at C3 and C4, approximately corresponding to the locations of the sensorimotor hand areas. Multiple additional movement and hand related features become apparent throughout the maps, manifest also in additional ANOVA effects (Table 1). Within the *δ/θ*-bands, superimposed on the standing distribution, are additional effects and interactions not present at rest due to the SEED/HAND and SEGMENT factors. For the frontal sites (Fig. 4A), while there were no significant main effects of SEED/HAND or SEGMENT, a trend interaction of HAND*Y*SEGMENT was obtained (*p* < .05), indicating movement and hand related changes in centro-frontal *δ*-coherence. For the parietal sites (Fig. 4B), there were no significant main effects or interactions involving the SEGMENT factor, but a highly significant HAND*X interaction appears (*p* < .001), indicating a hand-related skewing of the centro-parietal *δ−*coherence, which is consistent with the known crossed somatotopy of the cerebral sensorimotor representation. For the cerebellar sites (Fig. 4C), two-way Y*SEGMENT (*p* < .05) and three-way interactions HAND*Y*SEGMENT (*p* < .05) were obtained.

**Table 1:** ANOVA effects for Cz row seeds

SEED-TARGET			δ		θ		α		β		γ	
	FACTOR	df	F	*p*	F	*p*	F	*p*	F	*p*	F	*p*
Cz-FCz	HAND	1,5										
X range 7	X	6,30	6.2	< 0.05	5.6	< 0.05						
	Y	1,5			9.6	< 0.05			10.1	< 0.05		
	SEGMENT	15,75							4.7	< 0.05	2.7	= 0.071
	HAND^Y	2,10	5.1	= 0.073							4.6	= 0.083
	X^Y	6,30	3.01	= 0.08	8.5	< 0.01						
	X^SEGMENT	90,450							2.5	= 0.073		
	HAND^X^SEGMENT	90,450							2.9	< 0.05		
	HAND^Y^SEGMENT	15,75	4.9	< 0.05								
Cz –CPz	HAND	1,5										
X range 9	X	8,40	**12.0**	**< 0.005**	8.7	< 0.05						
	Y	2,10	11.2	< 0.05	**27.0**	**< 0.001**						
	SEGMENT	15,75	3.4	= 0.10								
	HAND^X	8,40	**14.8**	**< 0.001**	3.0	= 0.081						
	HAND^Y	2,10	5.2	= 0.064								
	X^Y	16,80	**6.0**	**= 0.005**	**15.4**	**< 0.001**						
	X^SEGMENT	120,600	3.5	= 0.058	2.4	= 0.10						
Cz -SIz	HAND	1,5										
X range 7	X	6,30	3.2	= 0.11	3.9	= 0.052						
	Y	2,10			5.0	= 0.073						
	SEGMENT	15,75							4.5	< 0.05		
	HAND^X	6,30			2.9	= 0.096						
	X^Y	12,60	5.0	< 0.05							2.7	= 0.081
	Y^SEGMENT	30,150	5.1	< 0.05	4.0	< 0.05						
	HAND^Y^SEGMENT	30,150	4.1	< 0.05								

#### Beta-band Movement Effects

**Fig. 5:**
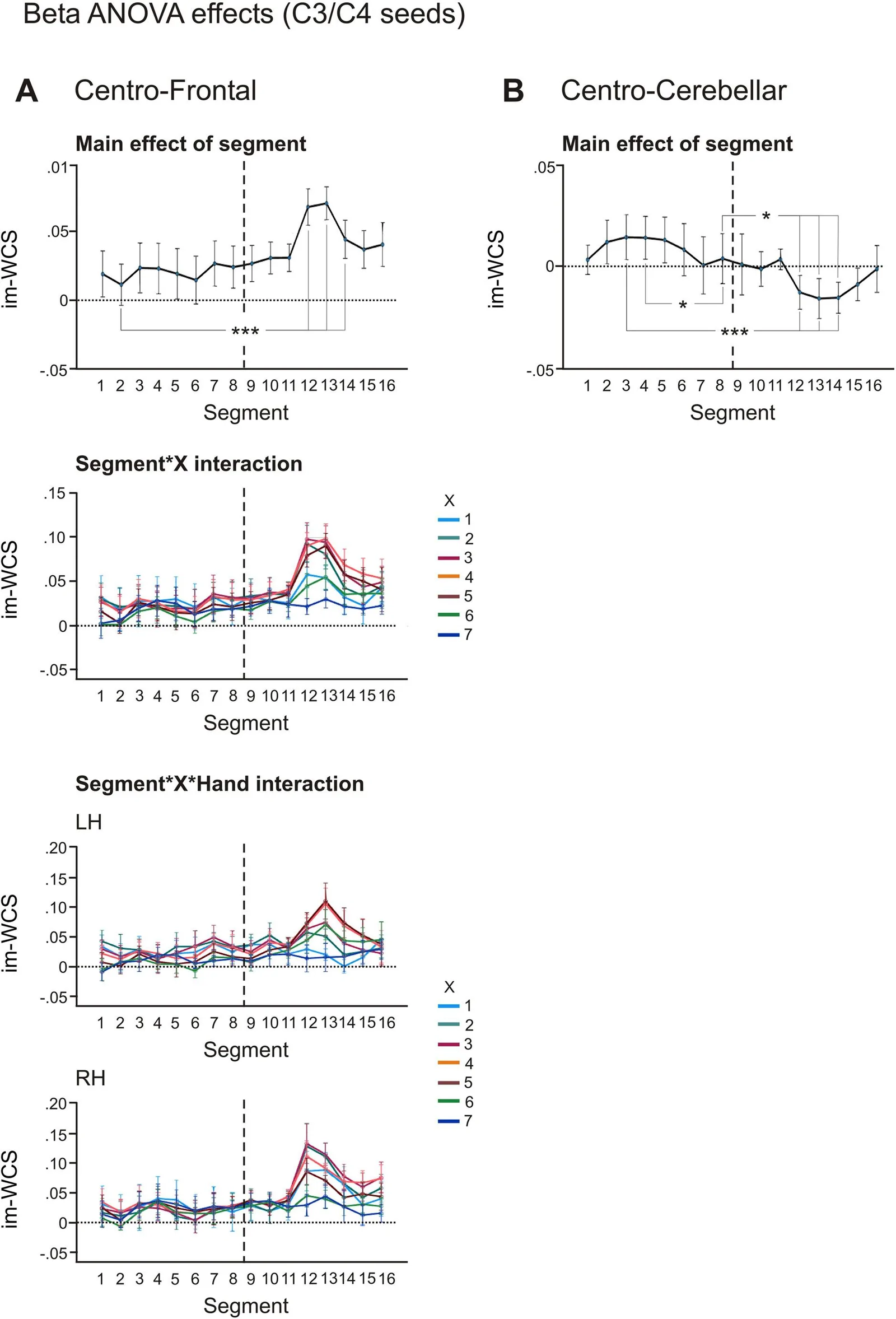
Changes in beta coherence with C3/4 seeds. ANOVA effects are shown for the changes in centro-frontal (**A**) and centro-cerebellar (**B**) coherence. In (A) X = 1–7 refers to the FC5, FC3, FC1, FCz, FC2, FC4 and FC6 columns. * *p* < .05, *** *p* < .005. Points represent estimated marginal means from the repeated-measures ANOVA. Error bars indicate ± 1 SEM; *n* = 6 participants

In addition to the low-frequency δ/θ-effects, *β*-band effects are also present in both the centro-frontal and centro-cerebellar coherence. For the frontal leads these are manifest in both a trend main effect of SEGMENT (*p* < .05) and a three-way interaction HAND*X*SEGMENT (*p* < .05) (Fig. 5A), consistent with the somatotopy of the cerebral sensorimotor representation, i.e. with right hand effects maximal in the left hemisphere (F1 column) and vice-versa (F2 column). For the cerebellar leads the *β*-band effect is primarily a trend main effect of SEGMENT (*p* < .05), with no spatial focus (Fig. 5B). For the centro-frontal *β*-effects, as well as being spatially focused with the expected somatotopy, they are dominated by post-movement segments, i.e. represent primarily a reafference, as illustrated by pair-wise comparison. The centro-cerebellar movement-related *β*-coherence in contrast appears to show pre- as well as post-movement changes, as supported by pair-wise comparison (Fig. 5B).

### Coherence Maps with Frontal Seeds F1/2

#### Seed Selection

**Fig. 6:**
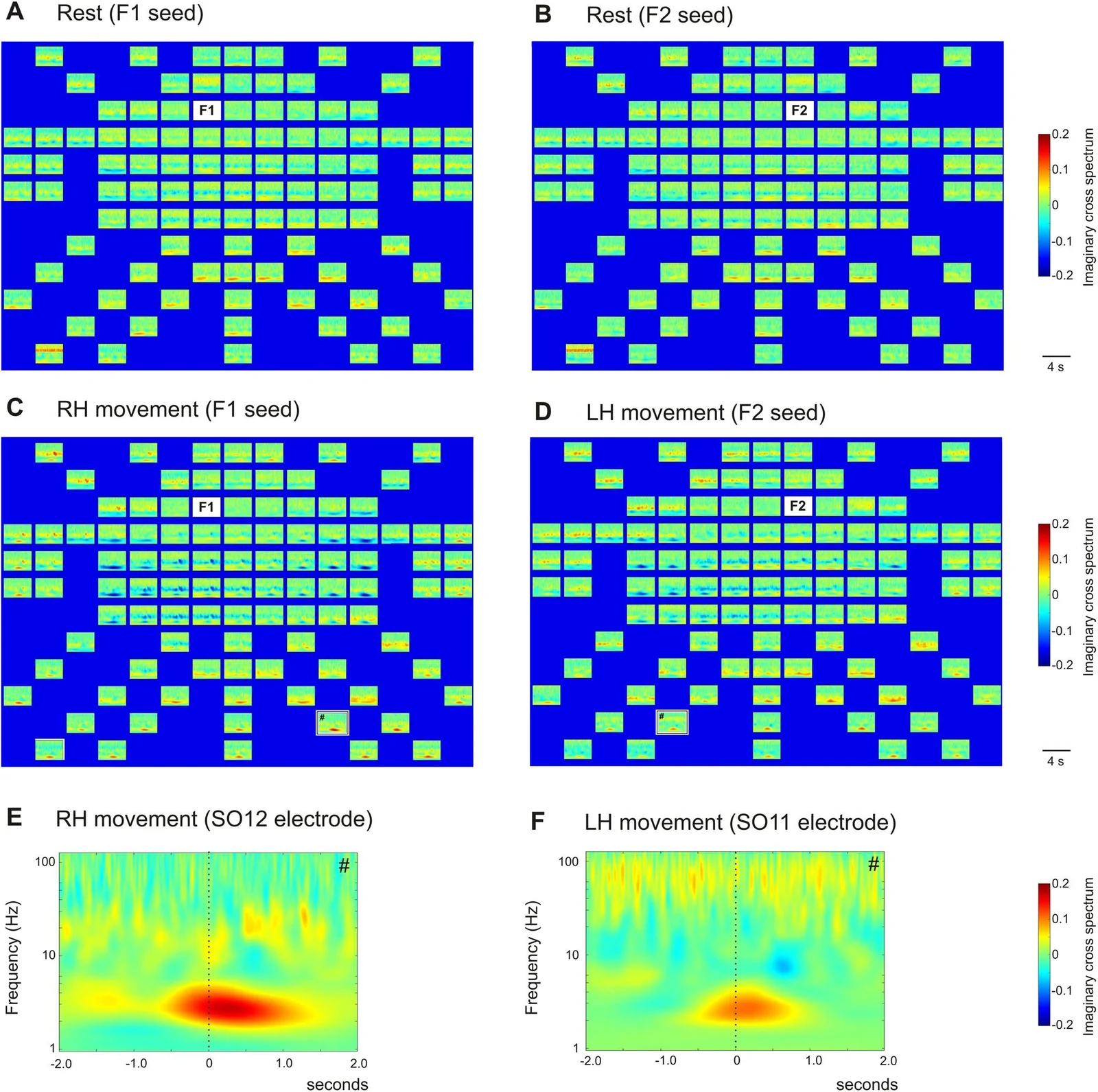
Coherence maps for F1 and F2 (frontal) seeds during rest (**A** & **B**) and movement (**C** & **D**)

**Fig. 7:**
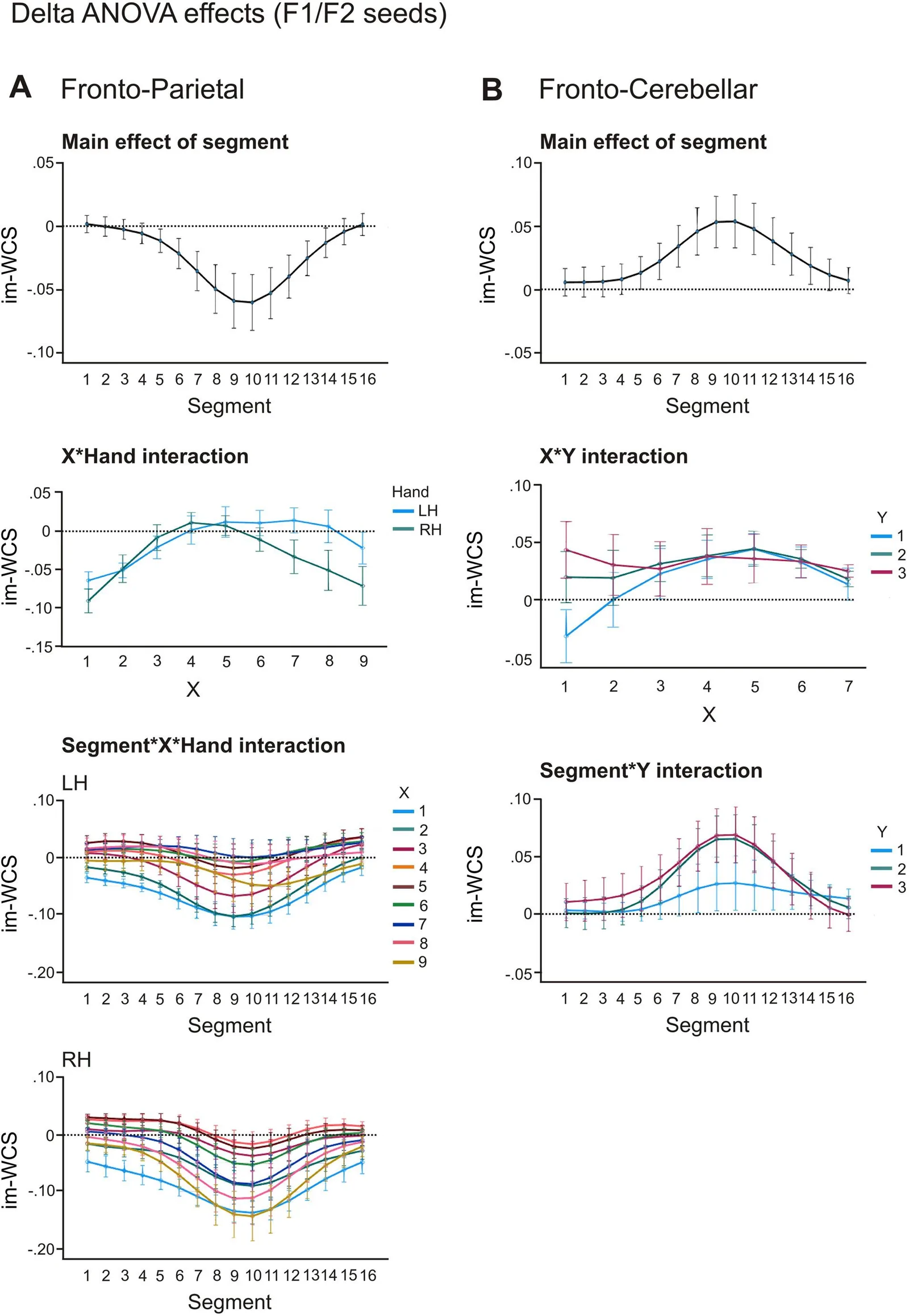
Changes in delta coherence with F1/2 seeds. ANOVA effects are shown for changes in fronto-parietal (**A**) and fronto-cerebellar (**B**) coherence. In (A) X = 1–9 refers to TP7, CP5, CP3, CP1, CPz, CP2, CP4, CP6 and TP8 columns; in (B) X = 1–7 refers to P11, PO11, SO11, SIz, SO12, PO12 and P12 columns and Y = 1–3 refers to the Iz, SIz and Bz rows. Points represent estimated marginal means from the repeated-measures ANOVA. Error bars indicate ± 1 SEM; *n* = 6 participants

**Fig. 8:**
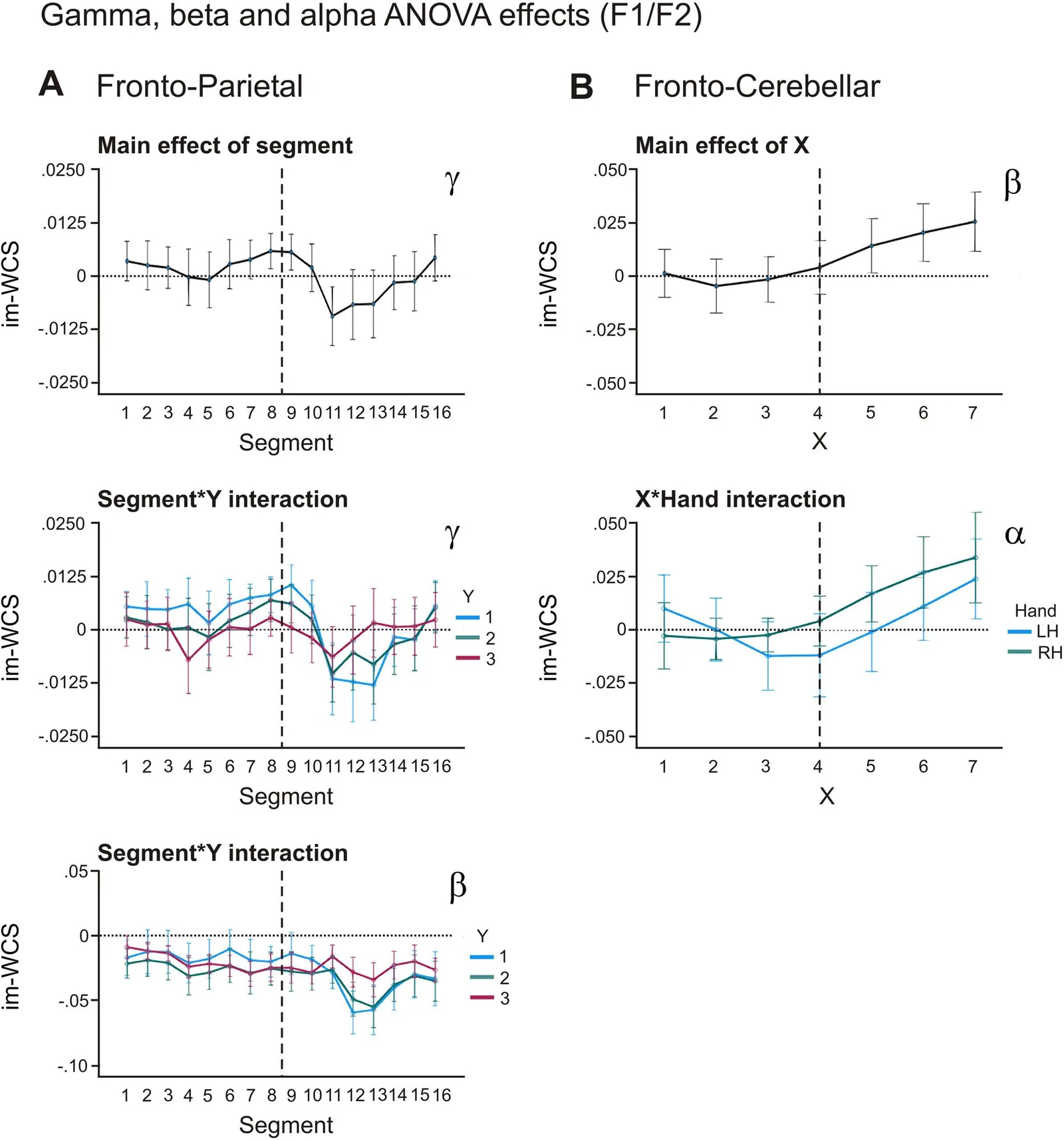
Changes in gamma, beta and alpha coherence with F1/2 seeds. ANOVA effects are shown for changes in fronto-parietal (**A**) and fronto-cerebellar (**B**) coherence. In (A) Y = 1–3 refers to the Cz, CPz and Pz rows; in (B) X = 1–7 refers to the P11, PO11, SO11, SIz, SO12, PO12 and P12 columns. Points represent estimated marginal means from the repeated-measures ANOVA. Error bars indicate ± 1 SEM; *n* = 6 participants

The C3/C4 seeded maps above revealed significant *δ/θ*- and *β*-band coherence which showed varied spatial and temporal distributions in frontal, parietal and cerebellar targets. In this section we report the properties of coherence maps seeded at F1 and F2 (Figs. 6, 7 and 8; Table 2), which were close to the spatial focus of the C3/C4 seeded *β*-coherence (Fig. 3E/F).

**Table 2:** ANOVA effects for Fz row seeds

SEED-TARGET			δ		θ		α		β		γ	
	FACTOR	df	F	*p*	F	*p*	F	*p*	F	*p*	F	*p*
Fz-CPz	HAND	1,5	6.0	= 0.058								
X range 9	X	8,40	5.3	< 0.05	7.6	< 0.05						
	Y	2,10			6.3	< 0.05						
	SEGMENT	15,75	9.3	< 0.05							3.4	< 0.05
	HAND^X	6,30	**13.3**	**= 0.001**								
	HAND^Y	2,10	7.7	< 0.05								
	X^Y	16,80	4.4	< 0.05	**16.6**	**< 0.001**						
	Y^SEGMENT	30,150							2.8	= 0.06	**4.7**	**= 0.005**
	HAND^X^SEGMENT	120,600	**6.8**	**= 0.001**								
Fz-SIz	HAND	1,5										
X range 7	X	6,30							6.3	< 0.05		
	Y	2,10			**45.7**	**< 0.001**						
	SEGMENT	15,75	**9.5**	**= 0.005**								
	HAND^X	6,30					7.1	< 0.01				
	HAND^Y	2,10			5.5	< 0.05						
	X^Y	12,60	3.4	= 0.065								
	Y^SEGMENT	30,150	5.0	< 0.05	2.9	= 0.07						
	HAND^Y^SEGMENT	30,150										

#### Delta-band Movement Effects

From visual inspection of the F1/F2 rest coherence maps (Fig. 6A/B), unlike the C3/C4 seeded map, there is no apparent low-frequency standing coherence either in parietal or cerebellar electrode grids. However, prominent movement-related δ/θ-coherence becomes apparent in both parietal and cerebellar sites for left and right handed movement (Fig. 6C/D). This is manifest in main effects of SEGMENT at both parietal and cerebellar sites in the parietal and cerebellar grids (respectively *p* < .05 and *p* = .005) (Fig. 7A/B), as well as interactions with SEED/HAND, X and Y for the parietal grid (*p* = .001), and with Y, but not X in the cerebellar grid (*p* < .05) (Table 2). The parietal three-way interaction is accompanied by a HAND*X interaction (*p* = .001), which indicates a right hand dominated skewing of the fronto-parietal *δ−*coherence, parallel to the centro-parietal effects above.

#### Alpha/beta/gamma-band Movement Effects

The fronto-cerebellar *δ−*coherence in contrast does not indicate any SEED/HAND effects, but *α/β*-band X and HAND*X effects are present indicating skewing to the right-hemisphere (Fig. 8B), with a trend X*Y *δ*-effect (*p* = .065; Fig. 6B, second row) supporting a skewing peak over the SO12 column. We also observed fronto-parietal higher frequency main effects of SEGMENT (*γ*-band, *p* < .05) and a SEGMENT*Y interaction (*γ*-band, *p* = .005, *β*-band, *p* = .06) (Fig. 8A; Table 2).

### Coherence Maps with Cerebellar Seeds SO11/12

#### Seed Selection

**Fig. 9:**
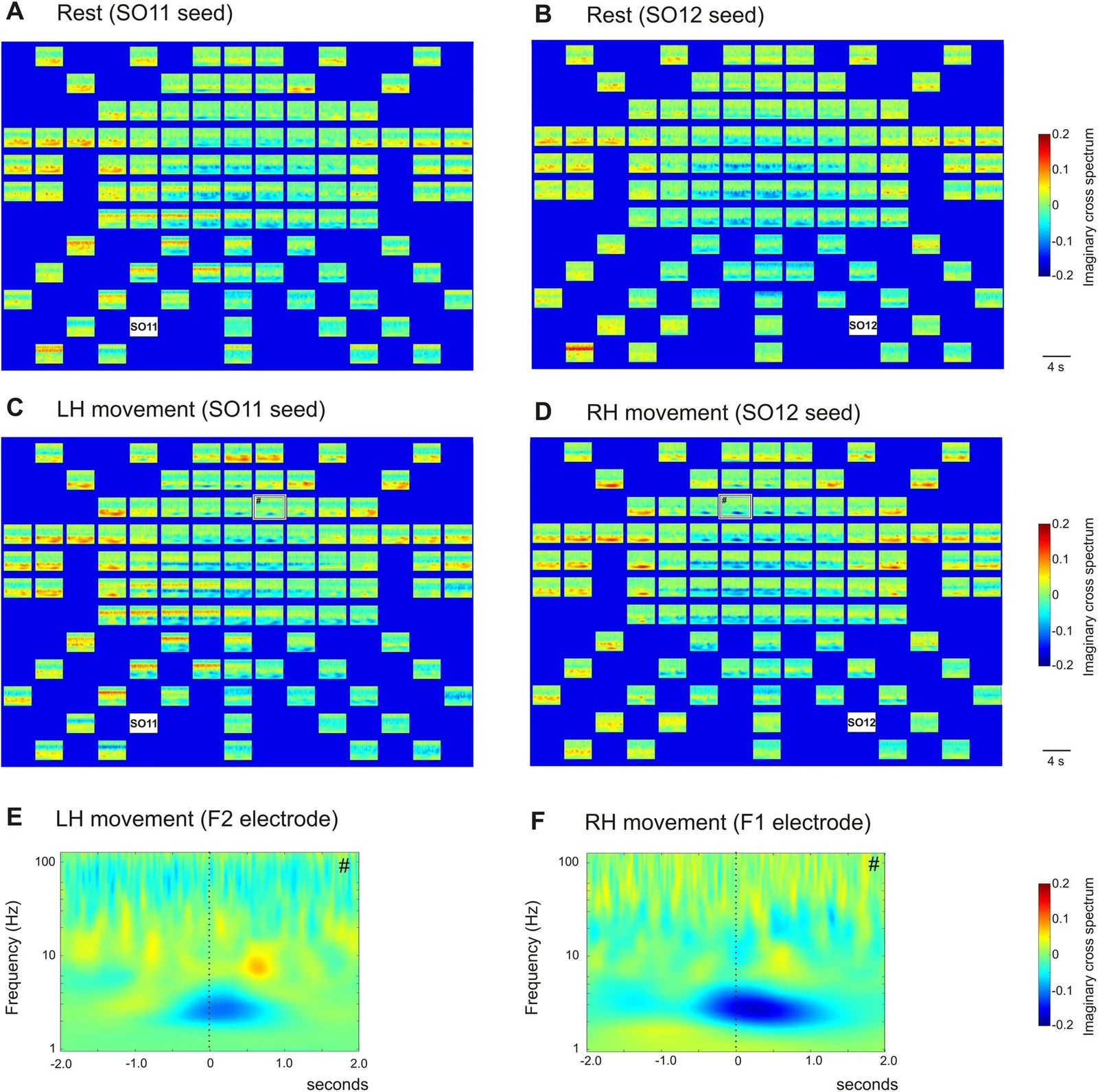
Coherence maps with cerebellar seeds (SO11/12) during rest (**A** & **B**) and movement (**C** & **D**). The bottom row shows single-electrode maps at the frontal sites; F2 (E; LH movement) and F1 (F; RH movement)

Both central and frontal seeds give rise to movement related *δ/θ*-coherence in the cerebellar grid in the form of either SEGMENT main effects or SEGMENT*Y interactions, suggesting foci within the SIz or Bz rows, i.e. at Y = 2 & 3, but with no strong X preference for selecting cerebellar seeds to investigate reciprocal cerebello-cerebral coherence. However, given the X*Y trend effect described above, we select SO12/SO11 as SEED locations for RH/LH respectively, which are also approximately where we would expect the posterior cerebellar hand representation to be located, crossed relative to the cerebral representation (Fig. 9).

#### Rest Effects

As with the central seeds, the SO12/SO11 seeds appear also to show a standing spatial distribution of *δ/θ*-coherence in frontal and parietal sites (Fig. 9A/B). This is apparent in a rest ANOVA with a *δ*-band X effect in frontal grid (*p* < .005), and *δ*-band X and Y effects in the parietal (respectively *p* < .001 and *p* < .005).

#### Delta/theta Movement Effects

**Fig. 10:**
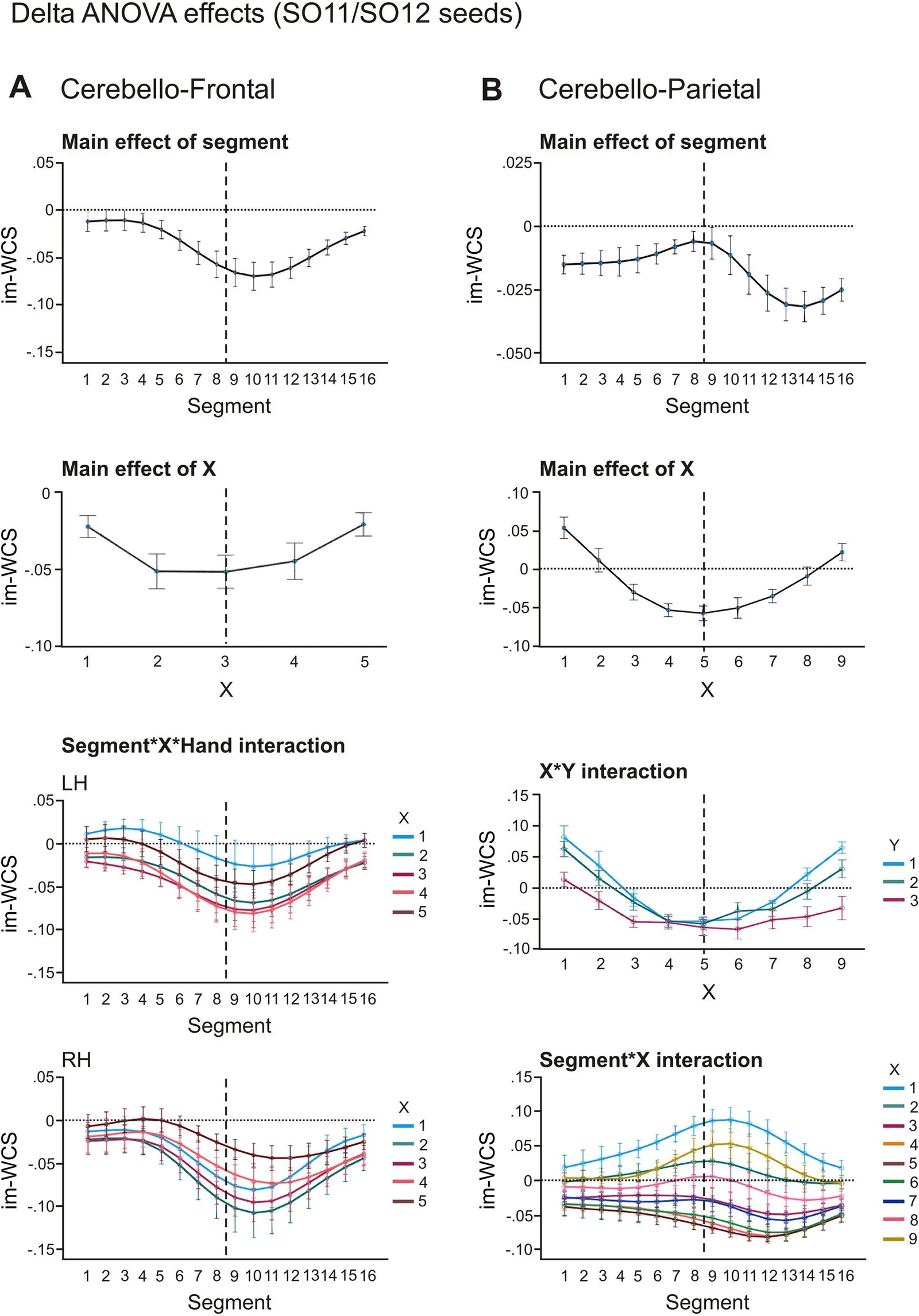
Changes in delta coherence with SO11/12 seeds. ANOVA effects are shown for changes in cerebello-frontal (**A**) and cerebello-parietal (**B**) coherence. In (A) X = 1–5 refers to the FC3, FC1, FCz, FC2 and FC4 columns; in (B) X = 1–9 refers to the TP7, CP5, CP3, CP1, CPz, CP2, CP4, CP6 and TP8 columns, Y = 1–3 refers to the Cz, CPz and Pz rows. Points represent estimated marginal means from the repeated-measures ANOVA. Error bars indicate ± 1 SEM; *n* = 6 participants

As with the C3/C4 case, hand movement induces additional ANOVA effects (Table 3) on top of the standing effects. For the frontal grid these are in the form of SEGMENT main effects (*p* < .01 and *p* = .005) and SEGMENT interactions with X, Y and HAND, depending on the X extent (+- 3 or +- 2) due to the midline focus (Table 3). If the X extent is restricted to +- 2 from the midline, the SEGMENT*X*HAND *δ*-band interaction (*p* < .05) is consistent with crossed cerebello-frontal projections (Fig. 10). For the parietal grid, movement-related *δ/θ*-coherence trend effects are also obtained in the form of SEGMENT main effects (respectively *p* = .07 and *p* < .05 for *δ*- and *θ*-bands), along with a SEGMENT^X interaction (*p* < .05) (Table 3). The *δ*-band SEGMENT main effect is partially cancelled by the change in sign of phase from –ve to +ve away from the midline, as is clear in the SEGMENT*X interaction, and indeed in the X main effect (*p* < .005) and X*Y effect (*p* = .001) which confirms the visual impression of strong movement-related *δ/θ*-coherence effects laterally, especially in the Cz and CPz rows (Y = 1, 2), i.e. at the T7/T8 and TP7/TP8 sites.

**Table 3:** ANOVA effects for SIz row seeds

SEED-TARGET			δ		θ		α		β		γ	
	FACTOR	df	F	*p*	F	*p*	F	*p*	F	*p*	F	*p*
SIz-FCz	HAND	1,5										
X range 7	X	6,30	**17.3**	**< 0.005**	**12.5**	**= 0.001**						
	Y	2,10			14.7	< 0.01						
	SEGMENT	15,75	9.4	< 0.01								
	X^SEGMENT	90,450	**6.0**	**= 0.005**								
	Y^SEGMENT	30,150			3.8	= 0.052			2.6	= 0.09		
	HAND^Y^SEGMENT	30,150	5.3	< 0.05								
SIz-FCz	HAND	1,5										
X range 5	X	4,20	**13.1**	**< 0.005**	6.1	< 0.05						
	Y	2,10			**26.8**	**< 0.005**						
	SEGMENT	15,75	**12.0**	**= 0.005**								
	HAND^X^SEGMENT	60,300	4.5	< 0.05								
	HAND^Y^SEGMENT	30,150	**8.2**	**< 0.005**								
SIz-CPz	HAND	1,5										
X range 9	X	8,40	**11.0**	**< 0.005**	7.0	< 0.05						
	Y	2,10	15.7	= 0.01	8.4	< 0.05						
	SEGMENT	15,75	4.4	= 0.07	4.4	< 0.05						
	HAND^X	8,40							3.5	= 0.067		
	X^Y	16,80	**8.3**	**= 0.001**	5.8	< 0.05						
	X^SEGMENT	120,600	5.8	< 0.05								
	Y^SEGMENT	30,150	3.6	= 0.055								

### Coherence Maps with Posterior Parietal Seeds TP7/TP8

#### Seed Selection

**Fig. 11:**
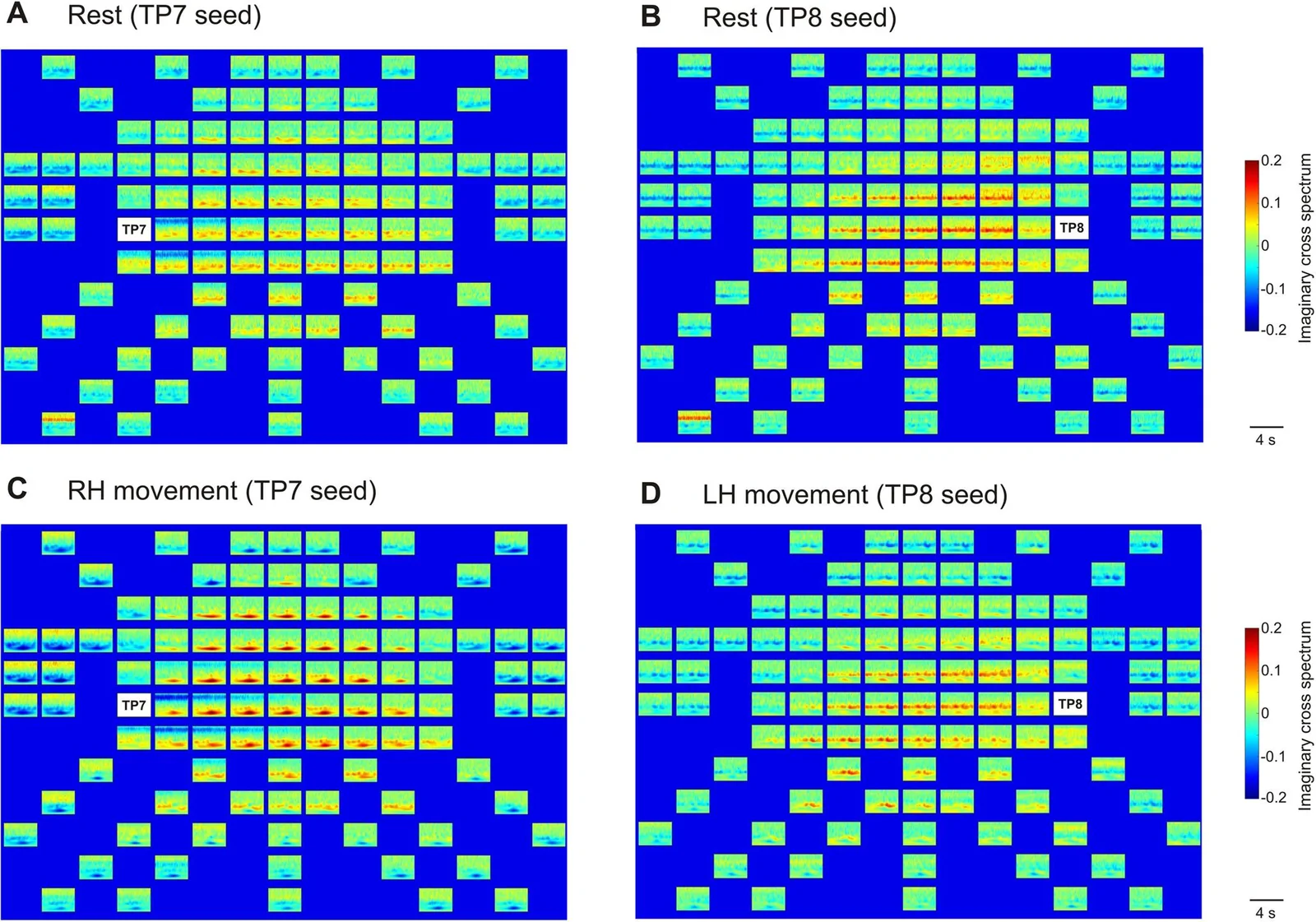
Coherence maps with parietal seeds (TP7/8) during rest (**A** & **B**) and movement (**C** & **D**)

**Fig. 12:**
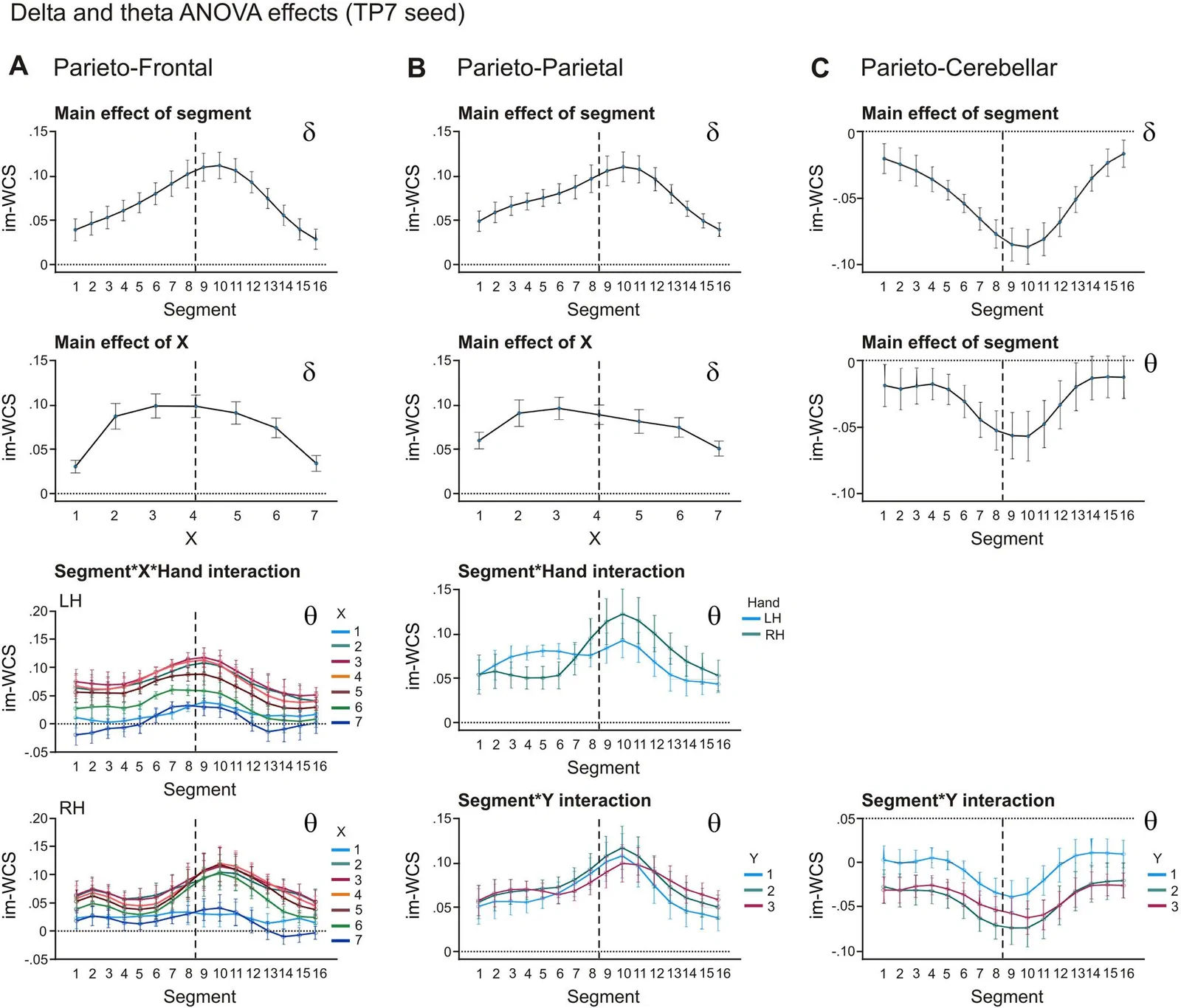
Delta and theta coherence changes with the TP7 seed. ANOVA effects are shown for changes in parieto-frontal (**A**; left column), parieto-parietal (**B**; middle column) and parieto-cerebellar (**C**; right column) coherence. In (A) X = 1–7 refers to the FC5, FC3, FC1, FCz, FC2, FC4 and FC6 columns; in (B) X = 1–7 refers to the CP5, CP3, CP1, CPz, CP2, CP4 and CP6 columns, Y = 1–3 refers to the Cz, CPz and Pz rows; in (C) Y = 1–3 refers to the Iz, SIz and Bz rows. Points represent estimated marginal means from the repeated-measures ANOVA. Error bars indicate ± 1 SEM; *n* = 6 participants

Given in particular the cerebello-parietal effects observed in "Coherence Maps with Cerebellar Seeds SO11/12" section, for completeness, we also generated the maps and ANOVA statistics with TP7/TP8 seeds (Figs. 11 and 12). However, given no prior obvious somatotopic lateralisation for the lateral posterior parietal sites, unlike the central, frontal and cerebellar sites, the hand asymmetry observed in 3.2 for the fronto-parietal coherence, and thus no clear basis to allocate a distinct handedness to a seed site, the statistics is based on the TP7 seed only for both left and right hand movement. There is thus no SEED factor. Visual inspection indicates strong parieto-parietal (latero-medial) effects and these are also included in the ANOVA results (Table 4).

**Table 4:** ANOVA effects for CPz row seeds

SEED-TARGET			δ		θ		α		β		γ	
	FACTOR	df	F	*p*	F	*p*	F	*p*	F	*p*	F	*p*
TP7-FCz	HAND	1,5										
X range 7	X	6,30	**35.7**	**< 0.001**	**24.7**	**< 0.001**						
	Y	2,10	9.4	< 0.05	21.6	= 0.001						
	SEGMENT	15,75	**13.8**	**< 0.005**	6.4	< 0.01						
	X^Y	12,60	3.3	= 0.051	**6.1**	**= 0.005**						
	X^SEGMENT	90,450	2.8	= 0.071								
	HAND^X^SEGMENT	90,450			3.1	< 0.05						
TP7-CPz	HAND	1,5									7.2	< 0.05
X range 7	X	6,30	**14.0**	**= 0.001**	**10.1**	**< 0.005**	4.7	< 0.05				
	Y	2,10					5.6	= 0.06				
	SEGMENT	15,75	**17.1**	**< 0.005**	5.2	< 0.05						
	HAND^X	6,30									5.0	= 0.063
	X^Y	12,60	3.2	= 0.066	6.0	< 0.01						
	HAND^SEGMENT	15,75			**6.4**	**< 0.005**						
	Y^SEGMENT	30,150			6.4	**= 0.005**						
TP7-SIz	HAND	1,5										
X range 7	X	6,30										
	Y	2,10	10.1	< 0.05	**23.3**	**< 0.001**			**12.3**	**< 0.005**		
	SEGMENT	15,75	**16.6**	**= 0.005**	**6.9**	**= 0.005**						

#### Delta/theta-band Movement Effects

All three grids at frontal, parietal and cerebellar sites show highly significant *δ*-band SEGMENT main effects (respectively, *p* < .005, *p* < .005 and *p* = .005) (Fig. 12A-C) and these extend to the *θ*-band (respectively, *p* < .01, *p* < .05 and *p* = .005). Highly significant *δ/θ*-band X-effects are also observed in frontal and parietal sites, consistent with a focus toward the midline. The frontal sites also yield a *θ*-band HAND*X*SEGMENT interaction (Fig. 12A) and a *θ*-band HAND*SEGMENT interaction (Fig. 12B) is observed at the parietal sites, indicative of right hand dominance. The cerebellar sites in contrast show no obvious HAND or X effects (Table 4).

### False Discovery Rate (FDR) Analysis

Supplementary Fig. 1 illustrates a FDR plot for all *p*-values presented in Tables 1, 2, 3 and 4 in seven lumped categories with FDR *q* = 0.05, 0.1 and 0.2 indicated. This analysis indicates that for effects with reported *p*-values < 0.001 there is low risk of a false positive at *q* = 0.05 or less. For effects with *p*-values between 0.001 and 0.01 type I error risk increases to between 0.1 and 0.2, but still remains sub-threshold for our exploratory study. For effects with *p*-values < 0.05 and > 0.01, most will become supra-threshold for *q* = 0.2 with the critical value of *p* = .019. As noted above, we will, therefore, treat such effects as trends. However, as we discuss below, it is important to consider the distribution of such trend effects among the ANOVA (spectral power, SEED/TARGET combination) and factor categories in order to avoid type II error. Of particular relevance, we note that within each of the seven lumped *p*-value categories the proportion which fall within the *δ/θ*-bands are respectively, 100% (*p* < .001), 100% (*p* = .001), 91% (*p* < .005), 88% (*p* = .005), 86% (*p* < .01), 50% (*p* = .01) and 79% (*p* < .05). Thus the proportion of the *p* < .05 category within the *δ/θ*-bands is four times greater than chance alone (20%). Even among the *p* > .05 tail the proportion within the *δ/θ*-bands is 47%, more than twice the chance rate.

### Evidence for Crossed Cerebro-cerebellar Connectivity in the Maps

**Fig. 13:**
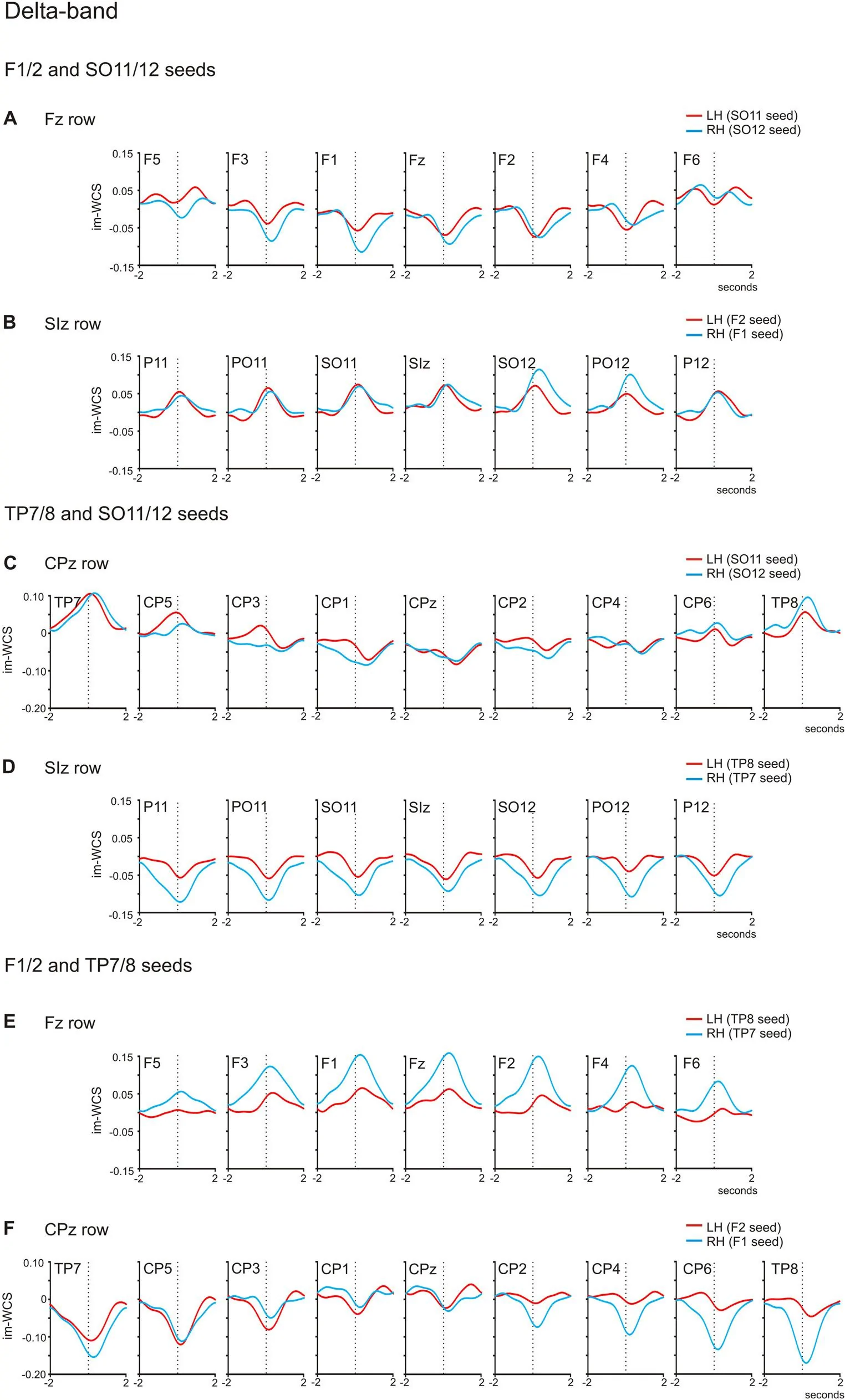
Delta-band coherence changes for multiple seed–electrode combinations. Panels A–F show delta-band waveforms (− 2 to 2 s) for left-hand (LH; red) and right-hand (RH; blue) movement conditions, plotted for different seed configurations and electrode rows. (A–B) F1/2 and SO11/12 seeds across the Fz row (**A**) and SIz row (**B**). (C–D) TP7/8 and SO11/12 seeds across the CPz row (**C**) and SIz row (**D**). (E–F) F1/2 and TP7/8 seeds across the Fz row (**E**) and CPz row (**F**)

The above four mapping analyses based at central, frontal, cerebellar and lateral parietal sites have shown predominantly (69%) *δ/θ*-band movement related SEGMENT effects, along with SEGMENT interactions with X, Y and HAND, consistent with a widespread *δ/θ*-band cerebro-cerebral and cerebro-cerebellar network for movement coordination. Of particular pertinence to the topic of cerebro-cerebellar connectivity, and for assessment of the validity of our proposed coherence mapping technique, is the question of what evidence is available within these effects for crossing in line with the known anatomy. For this purpose, we display in Fig. 13*δ*-band SEGMENT/HAND combinations for X and Y locations chosen using the SEED/HAND combinations employed for the maps.

The top row (Fig. 13A) shows the movement-related δ-band changes along the Fz row with the SO11/SO12 seed combination. For the SO12/RH combination the maximum SEGMENT effect (-ve phase) is observed at F1, while for the SO11/LH combination the maximum is observed at F2, and this is manifest as a HAND*X*SEGMENT interaction (*p* < .05, Table 3, “Coherence Maps with Cerebellar Seeds SO11/12” section). The reciprocal mapping, is illustrated underneath (Fig. 13B). For the F1/RH combination the maximum reciprocal (+ ve phase) effect is at SO12 (the phase inversion SO12 seeded RH image), while for the F2/LH combination there is no outstanding lateralised maximum and no significant corresponding interaction (Table 2, “Coherence Maps with Frontal Seeds F1/2” section).

The next row (Fig. 13C) shows the movement-related δ-band changes along the CPz row with SO11/SO12 seed combinations. In contrast to the cerebello-frontal projection, it is maximal away from the midline, manifest as an X*SEGMENT interaction (*p* < .05, Table 3, “Coherence Maps with Cerebellar Seeds SO11/12” section), with the maxima observed at TP7/8, though there are no SEED/HAND effects, also unlike the frontal map. As with the fronto-cerebellar case (Table 2, “Coherence Maps with Frontal Seeds F1/2” section), the parieto-cerebellar mapping shows neither X nor HAND interactions.

For completeness, we also illustrate the cerebral cortico-cortical reciprocal movement-related changes in frontal (F1/F2) and parietal seeded maps (TP7/8) (Fig. 13D-F). As with the previous two cases, the frontal grid shows a midline maximal X distribution and the parietal grid a lateral X distribution. However, both exhibit a mutual *δ*- or *θ*-band HAND*X*SEGMENT interaction, demonstrating a dominance of the right hand and with some evidence of left hemisphere dominance.

Considering the above evidence together, it is useful to collate the critical effects together, which we do in Table 5 for the *δ/θ*-band and Table 6 for the *α*/*β/γ*-band effects. From these we can see that *δ/θ*-band main X-effects are present at both cerebral target sites, irrespective of the seed site, indicative of consistent cerebral medio-lateral gradients, absent in the cerebellar sites. The frontal sites also consistently exhibit SEED/HAND interactions, irrespective of the seed site, also generally absent from the cerebellar target sites. The cerebellar target sites in contrast stand out as showing general *δ/θ*-band SEGMENT main effects with no other interactions, other than a Y-gradient with the C3/4 seeds.

**Table 5:** Summary of δ/θ-band movement-related coherence effects

Seed		Target Grid		
		FCz	CPz	SIz
C3/C4 (Table 1)	**Effects**	X HAND^X	X	Y^SEGMENT HAND^Y^SEGMENT
F1/F2 (Table 2)			X HAND^X HAND^X^SEGMENT	SEGMENT
TP7/8 (Table 4)		X SEGMENT HAND^X^SEGMENT	X SEGMENT HAND^SEGMENT	SEGMENT
SO11/12 (Table 3)		X SEGMENT HAND^X^SEGMENT	X SEGMENT X^SEGMENT	

**Table 6:** Summary of α/β/γ-band movement-related coherence effects

Seed		Target Grid		
		FCz	CPz	SIz
C3/C4 (Table 1)	**Effects**	Y SEGMENT HAND^X^SEGMENT		SEGMENT
F1/F2 (Table 2)			SEGMENT Y^SEGMENT	X HAND^X
TP7/8 (Table 4)			X HAND	Y
SO11/12 (Table 3)				

While HAND*X*SEGMENT interactions are absent in the F1/F2-seeded *δ/θ*-band coherence, this three-way interaction is uniquely present in the F1/F2-seeded *β/γ*-band coherence at the frontal sites (Table 6), which as we note above, is consistent with the established somatotopy of the cerebral sensorimotor representation. *β/γ*-band SEGMENT main effects are obtained in two other cases, i.e. within the cerebellar targets with C3/4 (motor) seeds, and within the parietal targets with F1/F2 (premotor) seeds.

## Discussion

In the present paper we have shown using non-invasive electrophysiology, with an extended 10% cerebellar montage, widely distributed movement-related changes in cerebro-cerebellar coherence, as well as cerebro-cerebral coherence. We have based this upon the imaginary component of coherence, to avoid volume-conducted and reference artefacts [39, 40]. Although the sample size was six, the principal effects observed were highly significant statistically. This is likely because the underlying Bereitschaftspotential (BPs) were individually present and measurable in all subjects [35], as demonstrated previously in source analyses of the same dataset [35]. Further analyses of the dataset have also demonstrated the feasibility of decoding cerebellar movement-related potentials at the level of individual participants [37]. For each of the subjects, we obtained up to 100 trials per condition (with maximum two to three lost trials in a few cases), and thus the signal to noise was robust. For this reason we have been able to conduct effectively several distinct analyses, including source analyses [35], spectral power analyses [36] and the present coherence analyses, the cerebral aspects of which are highly consistent with prior literature, thus supporting the validity of the analyses. We note also the very high preponderance of effects concentrated in the *δ/θ*-bands. At the strictest significance levels, 100% of our effects are concentrated in the *δ/θ*-bands. This provides clear evidence that our strongest signals are directly tied to the primary spectral components of the BP, thus further supporting the study validity. Even in the lower significance level effects the characteristic *δ/θ*-signature remains present. Our previously reported MRS power effects were also predominantly concentrated in the *δ/θ*-bands, along with cerebral *α/β*-MRD during self-paced voluntary movement [36].

The *δ/θ*-band cerebro-cerebellar movement-related coherence (MRC) reported here is likewise bi-directional, being observed both in cerebellar targets with cerebral seeds and in cerebral targets with cerebellar seeds. Its magnitude is of the same order as cerebro-cerebral *δ/θ*-MRC and is indicative of cerebellar participation in a distributed *δ/θ*-band network associated with the preparation and execution of voluntary movement. This interpretation is consistent with our prior source analysis results [35], and with the stage-dependent cerebro-cerebellar interactions reported recently by Romano et al. [42]. This perspective is strengthened by the observation of an apparent crossed interaction (i.e. the HAND*X*SEGMENT interaction) consistent with the known somatotopy and its dynamic manifestations. Cerebro-cerebellar *δ/θ*-MRC is observed as a modulation of standing or resting cerebro-cerebellar coherence which is consistent with prior evidence of cerebro-cerebellar connectivity. This is especially via the middle cerebellar peduncle which carries the bulk of the fibres of the corticopontocerebellar pathway [7]. The data also exhibited some asymmetries, as noted in “Coherence Maps with Frontal Seeds F1/2” section, which may be related to handedness.

Cerebral *δ/θ*-MRS associated with MRPs recorded by EEG has been well-described in the literature for both self-paced and triggered voluntary movements [43, 44]. More generally it is believed that *δ*-oscillations play a role in cognitive processing [45]. Delta power within the cerebellum has also been described, most notably in relation to slow-wave sleep, where it is believed that *δ*-oscillations play a role in the coordination of intracerebellar, cerebello-hippocampal and cerebello-cerebral interactions in sleep-dependent memory processes [46, 47]. Such sleep studies of cerebellar slow-wave activity have generally been conducted using invasive means in animal models as it had been believed that it was not possible to record this non-invasively [30]. These studies confirmed slow-wave interactions between motor cortex and cerebellum [15, 46]. Thus, it is plausible that *δ*-oscillations might also contribute to cerebro-cerebellar coordination during voluntary movement in the waking state [19].

In addition to the predominant *δ/θ*-MRC, we also observed some higher frequency effects, most obviously the centro-frontal interaction (i.e. the HAND*X*SEGMENT interaction) which is consistent with known cerebral dynamic somatotopy, and the reciprocal fronto-centroparietal effects. Although these higher frequency SEGMENT effects were sparser, they were not randomly distributed but clearly tied to a motor/premotor SEED signature. Their lower general significance levels compared to high-amplitude *δ/θ*-effects is to be expected given a 1/*f* attenuation in power. Movement related changes in *β*-power in the form of pre-movement MRD and post-movement rebound MRD over the predominantly contralateral cerebral sensorimotor areas have been very well-described, and are thought to play a critical role in sensorimotor coordination, but also extend to SMA, premotor and secondary somatosensory areas, as well as subcortically to thalamus and basal ganglia, and indeed to the musculature [48, 49]. Our observations of centro-fronto-parietal *β/γ*-MRC are thus more generally consistent with prior work and provide validatory evidence for our approach.

In addition to these well-established effects, we did also observe a more diffuse general centro-cerebellar *β*-MRC (Fig. 5B). Coherent local field potentials in the *α*/*β*/*γ*-range from within primate SI/MI and paramedian cerebellar cortex have been described during attentive rest [17]. *β/γ*-band coherence has also been described during movement [18], and may play a role in the cerebellar modulation of cerebral coherence [50]. MEG based source analysis confirms a beta connectivity between motor cortex and cerebellum in humans [48]. Tractography places the bulk of the cortico-ponto-cerebellar tracts as terminating in the hemispheres [7], but a projection from motor cortex to vermal VIIIA/B has been described [51]. Thus we may expect wide distribution of *β/γ*-band cerebro-cerebellar coherency. Of interest, the general centro-cerebellar *β*-MRC also showed a change in sign from the premovement to postmovement period (Fig. 5B), suggesting stage-dependent modulation of cerebro-cerebellar interactions consistent with the findings of Romano et al. [42].

Another consistent finding in the coherence mapping, although not analysed statistically, was that both resting and movement-related coherence was distributed anterior to the cerebellar hemispheres to inferior temporal and frontal temporal leads, suggestive of co-coherence with temporal lobe structures at rest and during movement. Our prior source analysis study consistently indicated sources within hippocampal, parahippocampal, inferior temporal and medial temporal areas during voluntary movement [35]. There has been a growing awareness that cerebello-hippocampal interactions may have wider function beyond slow-wave activity in sleep. This includes a wide range of behaviours, including in classical conditioning, voluntary movement and spatiotemporal processing [52]. These may be mediated by multiple bi-directional pathways, including ipsilateral direct subcortical connections [53]. The hippocampus and associated temporal lobe structures project widely to frontal and parietal cortices as well as contralaterally via the hippocampal commissure [54].

The properties of non-invasive cerebro-cerebellar coherence shown here suggest numerous applications in the further investigation of the wider role of the cerebellum in motor learning, cognition and affect. Given the fundamental relationship between action and cognition [55], the highly significant effects of cerebro-cerebellar *δ*-MRC and the wider role that *δ*-oscillations play in cognitive processing, *δ*-MRC and, more generally, event related delta coherence could be a powerful tool as a dependent variable in experimental design.

Given also the increasing recognition of the role of the cerebellum in cognitive and affective dysfunction, as well as of course in motor dysfunction, non-invasive cerebro-cerebellar coherence mapping could also have significant benefit for diagnosis. Examples of cognitive/affective dysfunction implicating the cerebellum include schizophrenia [56], cerebellar cognitive affective syndrome [57], ADHD [58], autism [29] and dyslexia [59]. In schizophrenia alterations in frontal *δ*-power have been demonstrated and benefits of delta-frequency cerebellar stimulation suggested [60]. The cerebellum has also been implicated in temporal lobe epilepsy, manifest in the form of *δ*/*θ*/*α*-activity in the EEG, via its connectivity with the hippocampus [52], and cerebellar stimulation has also been proposed to inhibit hippocampal seizures [52]. Altered motor-related cortical connectivity has also been reported following focal cerebellar lesions, suggesting widespread network reorganisation consequent to cerebellar dysfunction [61]. Ready and reliable non-invasive access to related patterns of cerebro-cerebellar interaction thus provides a valuable potential assessment tool for a wide range of clinical conditions. However, the extent of variability or noise in the EEG in these clinical populations, e.g. due to movement artefacts or the effects of medications, may limit the utility of application EEG-ECeG coherence analysis in single case diagnosis.

In another potential application, movement-related *δ*-power from over the cerebellum has been recently demonstrated as providing a plausible signal for brain-computer interfacing [37], a technology that exploits a direct communication link between the brain’s electrical activity and external devices. Cerebro-cerebellar coherence may likewise also provide a novel signal for such purposes. Clearly, however, given the exploratory nature of our analyses, any such potential applications will require confirmation in larger and more targeted experimental studies.

## Supplementary Information

Below is the link to the electronic supplementary material.

**Supplementary Figure 1**. A false discovery rate (FDR) plot (for 825 tests) illustrating all *p*-values (log scale) from Tables 1 – 4 versus p-value rank. All values < .05 are lumped into seven categories with a de-lumped interpolation line superimposed. Also illustrated are FDR threshold curves for *q* = 0.05, 0.1 and 0.2. Intersection of the *q* = 0.2 threshold curve with the *p*-value interpolation line close to *p* =.01 allows values <= .01 (ranks 1 – 41) to be classified as "significant exploratory discoveries". Values >.01 and < .05 (ranks 42 – 79) which fall above the *q* = 0.2 threshold curve are considered to be "trends", depending on biological plausibility for interpretation.

## Data Availability

Data and code available on reasonable request.
